# Risk of subsequent primary oral cancer in a cohort of 69,460 5-year survivors of childhood and adolescent cancer in Europe: the PanCareSurFup study

**DOI:** 10.1038/s41416-022-02016-w

**Published:** 2022-11-01

**Authors:** Ceren Sunguc, Michael M. Hawkins, David L. Winter, Isabelle M. Dudley, Emma J. Heymer, Jop C. Teepen, Rodrigue S. Allodji, Fabiën N. Belle, Francesca Bagnasco, Julianne Byrne, Edit Bárdi, Cécile M. Ronckers, Nadia Haddy, Thorgerdur Gudmundsdottir, Stanislaw Garwicz, Momcilo Jankovic, Helena J. H. van der Pal, Maja Česen Mazić, Christina Schindera, Desiree Grabow, Milena M. Maule, Peter Kaatsch, Melanie Kaiser, Brice Fresneau, Gisela Michel, Roderick Skinner, Thomas Wiebe, Carlotta Sacerdote, Zsuzsanna Jakab, Maria Winther Gunnes, Monica Terenziani, Jeanette F. Winther, Päivi M. Lähteenmäki, Lorna Zadravec Zaletel, Claudia E. Kuehni, Leontien C. Kremer, Riccardo Haupt, Florent de Vathaire, Lars Hjorth, Raoul C. Reulen

**Affiliations:** 1 Centre for Childhood Cancer Survivor Studies, Institute of Applied Health Research, University of Birmingham, Birmingham, UK; 2 Princess Máxima Center for Pediatric Oncology, Utrecht, The Netherlands; 3 Radiation Epidemiology Team, Center for Research in Epidemiology and Population Health, INSERM U1018, University Paris Saclay, Gustave Roussy, Villejuif, France; 4 Childhood Cancer Research Group, Institute of Social and Preventive Medicine, University of Bern, Bern, Switzerland; 5 Center for Primary Care and Public Health (Unisanté), University of Lausanne, Lausanne, Switzerland; 6 IRCCS Istituto Giannina Gaslini, Genova, Italy; 7 Boyne Research Institute, c/o no. 1, The Maples, Bettystown, Co Meath, A92 C635 Ireland; 8 St Anna Children’s Hospital, Vienna, Austria and Department of Paediatrics and Adolescent Medicine, Johannes Kepler University Linz, Kepler University Hospital, Linz, Austria; 9 Department of Health Services Research, Carl von Ossietzky University of Oldenburg, Oldenburg, Germany; 10 Danish Cancer Society Research Center, Childhood Cancer Research Group, Copenhagen, Denmark; 11 Children’s Hospital, Landspitali University Hospital, Reykjavik, Iceland; 12 Department of Clinical Sciences Lund, Paediatrics, Skane University Hospital, Lund University, Lund, Sweden; 13 Pediatric Clinic, University of Milano-Bicocca, Hospital San Gerardo, Via Donizetti, 33 Monza, Italy; 14 University Children’s Hospital Ljubljana, University Medical Centre Ljubljana, Ljubljana, Slovenia; 15 Division of Pediatric Oncology/Haematology, University Children’s Hospital Basel, University of Basel, Basel, Switzerland; 16 German Childhood Cancer Registry, Division of Childhood Cancer Epidemiology, Institute of Medical Biostatistics, Epidemiology and Informatics (IMBEI), Johannes-Gutenberg University Mainz, Mainz, Germany; 17 Childhood Cancer Registry of Piedmont, Cancer Epidemiology Unit, Department of Medical Sciences, University of Turin and CPO-Piemonte, AOU Città della Salute e della Scienza di Torino, Turin, Italy; 18 Department of Children and Adolescents Oncology, Gustave Roussy, F-94805 Villejuif, France; 19 Department of Health Sciences and Medicine, University of Lucerne, Lucerne, Switzerland; 20 Great North Children’s Hospital, Newcastle upon Tyne Hospitals NHS Foundation Trust, and Newcastle University Centre for Cancer, Newcastle University, Newcastle upon Tyne, UK; 21 Hungarian Childhood Cancer Registry, 2nd Department of Pediatrics, Semmelweis University, Budapest, Hungary; 22 Division of Paediatric and Adolescent Medicine, Oslo University Hospital Rikshospitalet, Oslo, Norway; 23 Department of Registration, Cancer Registry of Norway, Oslo, Norway; 24 Pediatric Oncology Unit, Fondazione IRCCS Istituto Nazionale dei Tumori, Milano, Italy; 25 Department of Clinical Medicine, Faculty of Health, Aarhus University and University Hospital, Aarhus, Denmark; 26 Department of Pediatrics and Adolescent Medicine, Turku University and Turku University Hospital, Turku, Finland; 27 Division of Radiotherapy, Institute of Oncology, Ljubljana, Slovenia; 28 Division of Pediatric Hematology/Oncology, Department of Paediatrics, University Children’s Hospital of Bern, University of Bern, Bern, Switzerland; 29 Emma Children’s Hospital Amsterdam UMC, location University of Amsterdam, Department of Pediatrics, Amsterdam, the Netherlands; 30 DOPO clinic, Division of Hematology/Oncology, IRCCS Istituto Giannina Gaslini, Genova, Italy

**Keywords:** Risk factors, Cancer epidemiology

## Abstract

**Background:**

Survivors of childhood cancer are at risk of subsequent primary malignant neoplasms (SPNs), but the risk for rarer types of SPNs, such as oral cancer, is uncertain. Previous studies included few oral SPNs, hence large-scale cohorts are required to identify groups at risks.

**Methods:**

The PanCareSurFup cohort includes 69,460 5-year survivors of childhood cancer across Europe. Risks of oral SPNs were defined by standardised incidence ratios (SIRs), absolute excess risks and cumulative incidence.

**Results:**

One hundred and forty-five oral SPNs (64 salivary gland, 38 tongue, 20 pharynx, 2 lip, and 21 other) were ascertained among 143 survivors. Survivors were at 5-fold risk of an oral SPN (95% CI: 4.4–5.6). Survivors of leukaemia were at greatest risk (SIR = 19.2; 95% CI: 14.6–25.2) followed by bone sarcoma (SIR = 6.4, 95% CI: 3.7–11.0), Hodgkin lymphoma (SIR = 6.2, 95% CI: 3.9–9.9) and soft-tissue sarcoma (SIR = 5.0, 95% CI: 3.0–8.5). Survivors treated with radiotherapy were at 33-fold risk of salivary gland SPNs (95% CI: 25.3–44.5), particularly Hodgkin lymphoma (SIR = 66.2, 95% CI: 43.6–100.5) and leukaemia (SIR = 50.5, 95% CI: 36.1–70.7) survivors. Survivors treated with chemotherapy had a substantially increased risk of a tongue SPN (SIR = 15.9, 95% CI: 10.6–23.7).

**Conclusions:**

Previous radiotherapy increases the risk of salivary gland SPNs considerably, while chemotherapy increases the risk of tongue SPNs substantially. Awareness of these risks among both health-care professionals and survivors could play a crucial role in detecting oral SPNs early.

## Introduction

Five-year survival rates after treatment for childhood cancer have increased over the last few decades and are now over 80% in Europe [1, 2]. Half a million people in Europe alone have a history of childhood cancer [1, 3] and up to two-thirds of childhood cancer survivors develop at least one long-term complication following treatment [4]. Developing a subsequent primary malignant neoplasm (SPN) is one of the most severe long-term health complications following childhood cancer [5–9]. Previous studies investigating the long-term risk of SPNs found that survivors of childhood cancer are at 5 to 10-fold risk of developing an SPN compared to the general population, particularly breast, genitourinary and digestive cancer [5, 6]. However, the magnitude of the risk for rarer types of SPNs, such as oral cancer, is unclear. Previous studies suggested that head and neck radiation, human papillomavirus (HPV) and haematopoietic stem cell transplantation (HSCT) increase the risk of oral cancer [10–13], but most, if not all, studies included few oral SPNs, with the largest study to date including only 27 oral SPNs [14]. In addition, few studies investigated subtypes of oral SPNs such as salivary gland or tongue SPNs [12, 14, 15]. Although oral SPNs are rare among childhood cancer survivors, it is likely to have a severe adverse impact on survivors’ health-related quality of life [10]. It is therefore crucial to identify groups at the highest risk of developing oral SPNs among survivors.

The principal objective of this large-scale Pan-European cohort study was to investigate the risks of developing oral SPNs, including SPNs of the salivary glands and the tongue, among 69,460 5-year survivors of childhood and adolescent cancer.

## Methods

### PanCare Childhood and Adolescent Cancer Survivor Care and Follow-up Studies

The PanCare Childhood and Adolescent Cancer Survivor Care and Follow-up Studies (PanCareSurFup) is a study across 12 European countries that set up the largest cohort of childhood and adolescent cancer survivors to date [1]. A principal aim was to investigate the risks of SPNs among five-year survivors of childhood and adolescent cancer [1, 3, 6, 16, 17]. The cohort consisted of 69,460 individuals diagnosed with cancer between 1940 and 2008 age 0–20 years and who survived at least 5 years. Thirteen institutions from 12 different European countries contributed data (Supplementary Table S.1). Data collection methodology and country-specific differences of cohort characteristics are described elsewhere [1].

### Childhood cancer classification

Classification systems for the primary site and morphology of childhood cancers varied by country, but to ensure compatibility all site and morphology codes for each tumour were converted into the 3^rd^ revision of the International Classification of Diseases for Oncology (ICD-O-3) using the International Association of Cancer Registries (IACR) Check and Conversion Program. ICD-O-3 codes were then classified into subcategories of childhood cancer by applying the International Classification of Childhood Cancer third edition [18, 19]. Further details can be found elsewhere [6, 16, 17].

### SPN ascertainment

For each SPN, the histology had to be different from the original childhood cancer to count as an SPN. Validation of each SPN was through pathology reports. Oral SPNs (lip, tongue, salivary glands, oral cavity, and pharynx) were defined according to the ICD version relevant to the year of the SPN diagnosis (Supplementary Table S.2) [20].

### Statistical analysis

Time-at-risk commenced at 5 years after the date of childhood cancer diagnosis and ended at the first occurrence of: loss-to-follow up, death, or study exit date. The study exit date differed for each sub-cohort (Supplementary Table S.1). Individuals who were lost-to-follow up were censored at the last known date alive and oral cancer free. Multiple oral SPNs per survivor were allowed in calculations where comparisons were made with the general population. Standardised incidence ratios (SIRs) were calculated as the observed number of oral SPNs over the expected number. Expected numbers were estimated by accumulating person-years at risk within country, sex, age (5-year bands), and calendar year (1-year bands) specific strata in the survivor cohort and then multiplying the person-years within each stratum by the corresponding stratum specific oral cancer incidence rate from the general population. General population incidence rates were obtained through the open-source Cancer Incidence in Five Continents [21] and the European Cancer Observatory [22]. Absolute excess risks (AERs) were calculated as the difference of the observed and expected number of oral cancers, divided by the total number of person-years at risk and multiplied by 100,000. To evaluate the simultaneous effect of the factors; sex, childhood cancer diagnosis, decade of childhood diagnosis, age at childhood diagnosis, attained age, follow-up time, chemotherapy, and radiotherapy, multivariable Poisson regression models were used to calculate adjusted relative risks (RRs). Models including attained age did not include follow-up time (and vice versa) due to collinearity. The RRs can here be interpreted as the ratio of SIRs adjusted for relevant covariates. The Nordic countries and Italian population-based cohorts were excluded from analyses involving radiotherapy and chemotherapy variables as no treatment data were available for the Nordic Countries and <70% for the Italian population-based cohort. Likelihood ratio tests were used to calculate *p* values for heterogeneity and linear trend where applicable. The cumulative incidence of developing an oral SPN as a function of attained age was calculated accounting for death as a competing risk [23]. For all analyses, a *p* value < 0.05 was considered statistically significant. Stata software version 17.0 was used for all analyses.

## Results

### Cohort characteristics

In total, 69,460 childhood cancer survivors were followed up for 1,264,634 person-years with 145 oral SPNs ascertained among 143 survivors. The earliest oral SPN occurred at 5 years and latest at 52 years after childhood cancer diagnosis. The mean and median age of an oral SPN was 34 and 32 years, respectively. The most common types of oral SPNs were malignancies of the salivary gland (*n* = 64) and tongue (*n* = 38) (Table 1). Oral SPNs occurred most frequently among survivors of leukaemia (*n* = 52; acute lymphoblastic = 47; acute myeloid = 3; other = 2), Hodgkin lymphoma (*n* = 18), soft-tissue sarcoma (*n* = 14) and bone sarcoma (*n* = 13) (Table 2).

**Table 1:** SIR and AER for development of specific subsequent primary oral cancers among all 5-years cancer survivors in the PanCareSurFup cohort (*N* = 64,460).

Type of SPN	Median age	Obs (%)	Exp	SIR (95% CI)	AER (95% CI)
Salivary gland	27.8	64 (44%)	3.7	17.2 (14.4–20.4)	4.8 (4.0–5.7)
Tongue	32.4	38 (26%)	6.4	5.9 (4.7–7.4)	2.5 (1.9–3.3)
Other oral cavity	35.3	21 (14%)	5.7	3.7 (2.7–5.0)	1.2 (0.8–1.8)
Pharynx	44.7	20 (14%)	9.4	2.1 (1.6–2.9)	0.8 (0.5–1.5)
Lip	23.0	2 (1.4%)	1.5	1.4 (0.5–3.6)	0.0 (0.0–1.7)
Overall	32.4	145 (100%)	29.2	5.0 (4.4–5.6)	9.2 (7.9–10.6)

*SPN* subsequent primary malignant neoplasm, *Obs* observed, *Exp* expected, *SIR* standardised incidence ratio, *CI* confidence interval, *AER* absolute excess risks.

**Table 2:** SIR, RR and AER for any subsequent primary oral cancer by different factors among all cancer survivors in the PanCareSurFup cohort.

Factor	Level	*N* (%)	Pyrs	Obs (%)	Exp	SIR (95% CI)	RR (95% CI)	AER (95% CI)
Overall		69,460 (100%)	1,264,634	145 (100%)	29.2	5.0 (4.4–5.6)		9.2 (7.9–10.6)
Sex^a^	Males	37,738 (54.3%)	676,132	89 (61.4%)	20.3	4.4 (3.6–5.4)	1.0 (ref)	10.2 (7.8–13.3)
	Females	31,722 (45.7%)	588,502	56 (38.6%)	8.9	6.3 (4.8–8.2)	1.1 (0.8–1.5)	8.0 (5.9–10.9)
	*p*_heterogeneity_					0.04	0.61	0.25
Type of childhood cancer^a^	Leukaemia	16,595 (23.9%)	257,776	52 (35.9%)	2.7	19.2 (14.6–25.2)	6.9 (3.4–13.8)	19.1 (14.4–25.5)
	Hodgkin lymphoma	6000 (8.6%)	97,380	18 (12.4%)	2.7	6.2 (3.9–9.9)	3.6 (1.7–7.9)	15.5 (9.0–26.9)
	Non-HL	3350 (4.8%)	61,167	5 (3.4%)	2.9	2.9 (1.2–6.9)	1.8 (0.6–5.2)	5.3 (1.4–20.5)
	CNS tumour	14,529 (20.9%)	261,527	10 (6.9%)	1.8	1.5 (0.8–2.7)	1.0 (ref)	1.2 (0.2–8.4)
	Neuroblastoma	3169 (4.6%)	61,639	3 (2.1%)	6.8	3.4 (1.1–10.4)	2.1 (0.6–8.1)	3.4 (0.7–17.1)
	Retinoblastoma	2578 (3.7%)	70,267	5 (3.4%)	0.9	3.0 (1.3–7.2)	2.9 (0.9–9.1)	4.8 (1.3–17.7)
	Wilms tumour	4756 (6.8%)	108,429	4 (2.8%)	1.7	2.0 (0.8–5.5)	1.3 (0.4–4.4)	1.9 (0.3–12.8)
	Bone Sarcoma	3147 (4.5%)	56,775	13 (9.0%)	2.0	6.4 (3.7–11.0)	4.5 (1.9–10.3)	19.3 (10.1–36.8)
	Soft tissue sarcoma	4501 (6.5%)	92,019	14 (9.7%)	2.0	5.0 (3.0–8.5)	3.8 (1.7–8.6)	12.2 (6.3–23.4)
	Other	10,472 (15.1%)	185,835	21 (14.5%)	2.8	4.1 (2.7–6.3)	3.2 (1.5–6.9)	8.5 (4.9–15.0)
	Unclassified	363 (0.5%)	–	–	–	–	–	–
	*p*_heterogeneity_					<0.001	<0.001	<0.001
Decade of childhood cancer diagnosis^a^	<1970	8993 (12.9%)	310,237	31 (21.4%)	16.7	1.9 (1.3–2.6)	1.0 (ref)	4.6 (2.2–9.9)
	1970–1979	13,479 (19.4%)	353,288	46 (31.7%)	7.6	6.0 (4.5–8.1)	1.8 (1.1–3.0)	10.9 (7.7–15.4)
	1980–1989	20,900 (30.1%)	399,362	47 (32.4%)	3.9	12.1 (9.1–16.1)	2.2 (1.2–3.9)	10.8 (7.9–14.7)
	1990–2008	26,088 (37.6%)	201,748	21 (14.5%)	1.1	19.3 (12.6–29.5)	2.4 (1.2–5.1)	9.9 (6.3–15.5)
	*p*_trend_					<0.001	0.02	0.12
Age at childhood cancer (years)^a^	0–3	22,013 (31.7%)	438,196	30 (20.7%)	6.2	4.8 (3.4–6.9)	1.0 (ref)	5.4 (3.5–8.5)
	4–7	14,846 (21.4%)	278,311	29 (20.0%)	4.9	5.9 (4.1–8.5)	1.2 (0.7–2.1)	8.7 (5.6–13.4)
	8–11	11,199 (16.1%)	209,284	39 (26.9%)	6.0	6.5 (4.8–8.9)	1.7 (0.9–3.1)	15.8 (10.9–22.9)
	12–20	21,402 (30.8%)	338,843	47 (32.4%)	12.1	3.9 (2.9–5.2)	1.2 (0.6–2.5)	10.3 (7.0–15.1)
	*p*_trend_					0.27	0.28	0.008
Attained age (years) (Relates to age at study exit)^a^	<20	15,405 (22.2%)	410,373	18 (12.4%)	1.1	16.1 (10.1–25.5)	1.0 (ref)	4.1 (2.5–6.7)
	20–29	18,877 (27.2%)	419,216	47 (32.4%)	2.6	18.0 (13.5–24.0)	1.1 (0.5–2.1)	10.6 (7.8–14.3)
	30–39	17,144 (24.7%)	262,126	28 (19.3%)	4.7	6.0 (4.1–8.7)	0.4 (0.1–1.0)	8.9 (5.7–13.9)
	40–49	10,969 (15.8%)	120,676	36 (24.8%)	9.3	3.9 (2.8–5.3)	0.3 (0.1–1.2)	22.1 (14.2–34.3)
	50+	7065 (10.2%)	52,243	16 (11.0%)	11.5	1.4 (0.9–2.3)	0.2 (0.0–0.9)	8.6 (1.5–49.2)
	*p*_trend_					<0.001	<0.001	<0.001
Time since 5-year survival (years)^b^	0–9	23,923 (34.4%)	565,883	36 (24.8%)	2.2	16.0 (11.5–22.2)	1.0 (ref)	6.0 (4.2–8.5)
	10–19	15,801 (22.7%)	370,881	39 (26.9%)	3.7	10.7 (7.8–14.6)	0.7 (0.4–1.1)	9.5 (6.7–13.5)
	20–29	16,102 (23.2%)	212,291	39 (26.9%)	7.2	5.4 (3.9–7.4)	0.4 (0.2–0.7)	15.0 (10.2–22.0)
	30+	13,634 (19.6%)	115,578	31 (21.4%)	16.1	1.9 (1.4–2.7)	0.2 (0.1–0.4)	12.9 (6.2–26.8)
	*p*_trend_					<0.001	<0.001	0.002
Radiotherapy for childhood cancer^c^	Yes	20,036 (51.2%)	471,519	95 (79.2%)	13.1	7.2 (5.9–8.9)	2.6 (1.5–4.5)	17.4 (13.8–21.9)
	No	14,357 (36.7%)	263,552	18 (15.0%)	6.3	2.9 (1.8–4.5)	1.0 (ref)	4.4 (2.2–9.0)
	Unknown	4755 (12.1%)	–	7 (5.8%)	–	–	–	–
	Excluded^d^	30,312		25				
	*p*_heterogeneity_					<0.001	<0.001	<0.001
Chemotherapy for childhood cancer^c^	Yes	23,972 (61.2%)	448,030	87 (72.5%)	6.9	12.6 (10.2–15.6)	2.4 (1.3–4.3)	17.9 (14.2–22.5)
	No	10,335 (26.4%)	275,190	27 (22.5%)	11.8	2.3 (1.6–3.3)	1.0 (ref)	5.5 (2.8–10.8)
	Unknown	4841 (12.4%)	–	6 (5.0%)	–	–	–	–
	Excluded^d^	30,312		25				
	*p*_heterogeneity_					<0.001	0.004	<0.001

*Pyrs* person-years, *Obs* observed, *Exp* expected, *SIR* standardised incidence ratio, *RR* relative risk, *AER* absolute excess risks, *HL* Hodgkin lymphoma.

^a^RRs were derived from a model including sex, childhood cancer diagnosis, country, decade of childhood diagnosis, age at childhood diagnosis, and attained age.

^b^RRs were derived from a model including sex, childhood cancer diagnosis, country, decade of childhood diagnosis, age at childhood diagnosis, and follow-up time.

^c^RRs were derived from a model including sex, country, age at childhood diagnosis, attained age, and treatment.

^d^Excluded Nordic countries (Denmark, Sweden, Norway, Finland, Iceland) and Italy population-based data because of lack of treatment data.

### Risk of subsequent primary oral cancer

Overall, survivors were five times more likely than expected to develop an oral SPN (SIR = 5.0, 95% CI: 4.4–5.6) with 9 additional cases per 100,000 person-years (AER = 9.2, 95% CI: 7.9–10.6) (Table 2). Leukaemia survivors were at greatest risk with a 19.2-fold SIR (95% CI: 14.6–25.2). Although to a much lesser extent, survivors of bone sarcoma (SIR = 6.4, 95% CI: 3.7–11.0), Hodgkin lymphoma (SIR = 6.2, 95% CI: 3.9–9.9) and soft-tissue sarcoma (SIR = 5.0, 95% CI: 3.0–8.5) were also at high risk relative to the general population.

**Fig. 1:**
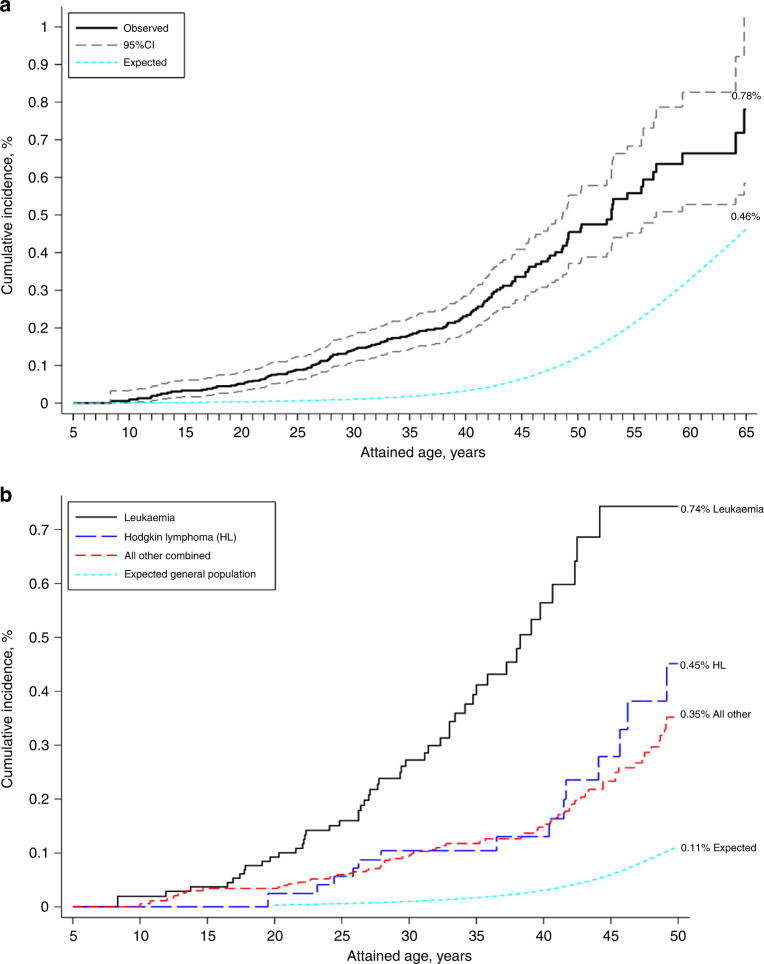
The cumulative incidence of developing a subsequent primary oral cancer as a function of attained age illustrated in all childhood cancer survivors (**a**) and in survivors of different types of childhood cancer (**b**).

Regarding attained age, the highest SIR was observed among those aged 20–29 years (SIR = 18, 95% CI: 13.5–24.0) and decreased thereafter with increasing attained age, however, the SIR was still elevated after age 40–49 years (SIR = 3.9, 95% CI: 2.8–5.3). Similarly, the SIRs also decreased with increasing time since 5-year survival (*p*_trend_ < 0.001). In contrast, the AERs increased with increasing attained age (*p*_trend_ < 0.001) and time since 5-year survival (*p*_trend_ < 0.001) due to an increase in the background incidence rate with increasing attained age and time since 5-year survival leading to higher expected numbers. By age 30 years, the cumulative incidence of developing an oral SPN was 0.14% (95% CI: 0.11–0.18%) for all survivors combined (Fig. 1a). By age 50 years, it was 0.45% (95% CI: 0.37–0.55%) and reached 0.78% (95% CI: 0.58–1.03%) by age 65 years (expected = 0.46%). By age 45 years, the cumulative incidence among leukaemia survivors was highest of all survivors reaching 0.74% while the expected incidence was only 0.06% (Fig. 1b), followed Hodgkin lymphoma survivors with a cumulative incidence of 0.28%.

Specific analyses for leukaemia survivors suggested that those diagnosed between 1990-2008 had a 53-fold risk of an oral SPN (SIR = 52.7, 95% CI: 31.8–87.4) (Table 3); however, multivariable analyses suggested that this was largely due to confounding, particularly by attained age, as there was no trend in RRs by decade of diagnosis in multivariable analyses. Survivors aged <30 years experienced the greatest SIRs at over 30-fold expected; however, the SIR was still increased 5.3-fold beyond age 40 years (95% CI: 2.4–11.8). The AER among leukaemia survivors increased significantly with attained age, reaching 37 beyond age 40 years (AER = 36.9, 95% CI: 13.7–98.9).

**Table 3:** SIR, RR and AER for any subsequent primary oral cancer by different factors in leukaemia survivors in the PanCareSurFup cohort.

Factor	Level	*N* (%)	Pyrs	Obs (%)	Exp	SIR (95% CI)	RR (95% CI)	AER (95% CI)
Leukaemia		16,595 (100%)	257,776	52 (100%)	2.7	19.2 (14.6–25.2)		19.1 (14.4–25.5)
Type of leukaemia	Acute lymphoblastic	14,538 (87.6%)	230,743	47 (90%)	2.4	19.7 (14.8–26.2)	–	19.2 (14.2–26.0)
	Acute myeloid	1449 (8.7%)	19,468	3 (6%)	0.2	12.9 (4.2–40.1)	–	14.2 (4.2–48.2)
	Other	608 (3.7%)	7565	2 (4%)	0.1	14.5 (3.6–58.1)	–	24.4 (5.5–107.4)
	*p*_heterogeneity_					0.68		0.84
Sex^a^	Males	8964 (54.0%)	133,457	30 (58%)	1.7	18.1 (12.6–25.9)	1.0 (ref)	21.2 (14.5–31.0)
	Females	7631 (46.0%)	124,319	22 (42%)	1.1	20.9 (13.7–31.7)	1.0 (0.6–1.7)	16.8 (10.9–26.1)
	*p*_heterogeneity_					0.6	0.99	0.43
Decade of childhood cancer diagnosis^a^	<1970	328 (2.0%)	7158	3 (6%)	0.3	10.9 (3.5–33.9)	1.6 (0.4–6.0)	38.1 (11.0–132.3)
	1970–1979	3138 (18.9%)	76,999	16 (31%)	1.3	12.6 (7.7–20.6)	1.0 (ref)	19.1 (11.2–32.6)
	1980–1989	5825 (35.1%)	112,434	18 (35%)	0.9	20.4 (12.8–32.3)	0.9 (0.4–1.9)	15.2 (9.4–24.7)
	1990–2008	7304 (44.0%)	61,185	15 (29%)	0.3	52.7 (31.8–87.4)	2.1 (0.9–5.0)	24.1 (14.4–40.3)
	*p*_trend_					<0.001	0.28	0.48
Age at childhood cancer (years)^a^	0–3	6715 (40.5%)	107,925	17 (33%)	0.8	22.5 (14.0–36.2)	1.0 (ref)	15.1 (9.2–24.8)
	4–7	5301 (31.9%)	85,782	15 (29%)	0.9	17.5 (10.5–29.0)	0.9 (0.4–1.8)	16.5 (9.6–28.2)
	8–11	2369 (14.3%)	36,200	11 (21%)	0.6	19.8 (11.0–35.7)	1.2 (0.5–2.9)	28.9 (15.5–53.8)
	12–20	2210 (13.3%)	27,870	9 (17%)	0.5	16.6 (8.6–31.8)	1.1 (0.4–3.4)	30.3 (15.1–60.8)
	*p*_trend_					0.46	0.60	0.06
Attained age (years)^a^	<20	5561 (33.5%)	116,587	10 (19%)	0.3	32.8 (17.7–61.0)	1.0 (ref)	8.3 (4.4–15.8)
	20–29	4602 (27.7%)	85,517	21 (40%)	0.6	37.9 (24.7–58.1)	1.3 (0.5–3.7)	23.9 (15.4–37.1)
	30–39	4090 (24.6%)	42,473	15 (29%)	0.8	19.5 (11.7–32.3)	0.7 (0.1–3.3)	33.5 (19.7–57.1)
	40+	2342 (14.1%)	13,200	6 (12%)	1.1	5.3 (2.4–11.8)	0.2 (0.0–1.7)	36.9 (13.7–98.9)
	*p*_trend_					<0.001	0.032	<0.001
Time since 5-year survival (years)^b^	0–9	6479 (39.0%)	130,655	15 (29%)	0.4	36.4 (21.9–60.3)	1.0 (ref)	11.2 (6.6–18.8)
	10–19	3995 (24.1%)	79,456	19 (37%)	0.6	30.4 (19.4–47.7)	0.9 (0.5–2.0)	23.1 (14.5–36.8)
	20–29	4216 (25.4%)	37,931	13 (25%)	0.9	14.8 (8.6–25.6)	0.5 (0.2–1.2)	32.0 (17.8–57.3)
	30+	1905 (11.5%)	9734	5 (10%)	0.8	6.2 (2.6–15.0)	0.2 (0.0–0.6)	43.1 (15.2–122.5)
	*p*_trend_					<0.001	<0.001	0.003
Radiotherapy for childhood cancer^c,d^	Yes	6846 (64.7%)	147,418	40 (87%)	1.8	22.5 (16.5–30.7)	4.4 (1.4–13.9)	25.9 (18.7–35.9)
	No	3542 (33.5%)	44,497	4 (9%)	0.5	8.5 (3.2–22.7)	1.0 (ref)	7.9 (2.6–24.1)
	Unknown	198 (1.9%)	–	2 (4%)	–	–	–	–
	*p*_heterogeneity_					0.03	0.006	0.02
Chemotherapy for childhood cancer^c,d^	Yes	10,570 (99.8%)	194,489	46 (100%)	2.3	20.3 (15.2–27.1)	–	22.5 (16.6–30.5)
	No	16 (0.2%)	221	0 (0%)	–	–	–	–
	*p*_heterogeneity_							

*Pyrs* person-years, *Obs* observed, *Exp* expected, *SIR* standardised incidence ratio, *RR* relative risk, *AER* absolute excess risks, *CI* confident interval.

^a^RRs were derived from a model including sex, childhood cancer diagnosis, country, decade of childhood diagnosis, age at childhood diagnosis, and attained age.

^b^RRs were derived from a model including sex, childhood cancer diagnosis, country, decade of childhood diagnosis, age at childhood diagnosis, and follow-up time.

^c^RRs were derived from a model including sex, country, age at childhood diagnosis, attained age and treatment.

^d^Excluded Nordic countries (Denmark, Sweden, Norway, Finland, Iceland) and Italy population-based data because of lack of treatment data.

### Risk of salivary gland cancer

The most common oral SPN was that of the salivary glands with 44% of all SPNs (*N* = 64) (Table 4). The overall SIR of developing a salivary gland SPN was 17.2 (95% CI: 14.4–20.4). SIRs were greatest among survivors of leukaemia (SIR = 40.1, 95% CI: 26.4–60.9) and Hodgkin lymphoma (SIR = 35.9, 95% CI: 20.4–63.2). Compared to the general population, survivors treated with radiotherapy were at 33-fold increased risk (SIR = 33.5, 95% CI: 25.3–44.5), particularly survivors of Hodgkin lymphoma (SIR = 66.2, 95% CI: 43.6–100.5), leukaemia (SIR = 50.5, 95% CI: 36.1–70.7) and CNS tumour (16.3, 95% CI: 8.7–30.2).

**Table 4:** SIR, RR and AER for subsequent primary malignant neoplasms (SPNs) of the salivary glands by different factors among all cancer survivors in the PanCareSurFup cohort.

SPN of salivary glands	Level	Obs (%)	Exp	SIR (95% CI)	RR (95% CI)		AER (95% CI)
	Overall	64 (100%)	3.7	17.2 (14.4–20.4)			4.8 (4.0–5.7)
Sex^a^	Male	34 (53%)	1.8	18.5 (13.2–25.9)	1.0 (1.0–1.0)		4.8 (3.3–6.8)
	Female	30 (47%)	1.9	15.9 (11.1–22.7)	0.9 (0.5–1.4)		4.8 (3.3–7.0)
	*p*_heterogeneity_			0.54	0.58		0.99
Type of childhood cancer^a^	Leukaemia	22 (34%)	0.5	40.1 (26.4–60.9)	4.4 (1.7–11.3)		8.3 (5.4–12.8)
	Leukaemia + RT*	17 (27%)	0.3	50.5 (36.1–70.7))	–		–
	Hodgkin lymphoma	12 (19%)	0.3	35.9 (20.4–63.2)	4.6 (1.7–12.5)		12.0 (6.7–21.4)
	Hodgkin lymphoma + RT*	11 (17%)	0.2	66.2 (43.6–100.5)	–		–
	Non-HL	5 (8%)	0.2	25.4 (10.6–61.1)	3.1 (0.9–10.2)		7.9 (3.2–19.6)
	CNS tumour	6 (9%)	0.8	7.1 (3.2–15.9)	1.0 (ref)		2.0 (0.8–5.0)
	CNS tumour + RT*	5 (8%)	0.3	16.3 (8.7–30.2)	–		–
	Neuroblastoma	1 (2%)	0.1	7.8 (1.1–55.0)	1.0 (0.1–8.5)		1.4 (0.1–13.4)
	Retinoblastoma	1 (2%)	0.2	5.1 (0.7–35.9)	0.8 (0.1–6.7)		1.1 (0.1–13.1)
	Wilms tumour	2 (3%)	0.3	7.8 (2.0–31.3)	1.1 (0.2–5.6)		1.6 (0.3–7.9)
	Bone sarcoma	4 (6%)	0.2	18.5 (6.9–49.3)	2.8 (0.8–9.9)		6.7 (2.4–18.8)
	Soft tissue sarcoma	6 (9%)	0.3	20.0 (9.0–44.4)	2.9 (0.9–9.0)		6.2 (2.7–14.4)
	Other	5 (8%)	0.7	7.4 (3.1–17.7)	1.3 (0.4-4.4)		2.3 (0.8–6.4)
	*p*_heterogeneity_			<0.001	0.003		<0.001
Decade of childhood cancer diagnosis^a^	<1970	15 (23%)	1.5	9.9 (6.0–16.4)	1.0 (ref)		4.3 (2.5–7.6)
	1970–1979	13 (20%)	1.0	13.3 (7.7–22.9)	0.7 (0.3–1.5)		3.4 (1.9–6.1)
	1980–1989	19 (30%)	0.9	22.1 (14.1–34.7)	0.8 (0.3–1.8)		4.5 (2.8–7.3)
	1990–2008	17 (27%)	0.4	45.7 (28.4–73.4)	1.6 (0.5–5.1)		8.2 (5.1–13.4)
	*p*_trend_			<0.001	0.31		0.13
Age at childhood cancer (years)^a^	0–3	14 (22%)	0.9	15.4 (9.1–26.0)	1.0 (ref)		3.0 (1.7–5.2)
	4–7	14 (22%)	0.7	20.1 (11.9–33.9)	1.1 (0.5–2.3)		4.8 (2.8–8.3)
	8–11	16 (25%)	0.7	23.1 (14.2–37.7)	1.4 (0.6–3.1)		7.3 (4.4–12.2)
	12–21	20 (31%)	1.4	14.0 (9.0–21.7)	1.1 (0.5–2.7)		5.5 (3.4–8.8)
	*p*_trend_			0.74	0.69		0.06
Attained age (years)^a^	<20	14 (22%)	0.4	35.3 (20.9–59.6)	1.0 (ref)		3.3 (1.9–5.7)
	20–29	22 (34%)	0.9	24.7 (16.3–37.5)	0.7 (0.3–1.5)		5.0 (3.3–7.8)
	30–39	11 (17%)	0.9	12.1 (6.7–21.9)	0.4 (0.2–1.0)		3.9 (2.0–7.3)
	40–49	12 (19%)	0.8	15.3 (8.7–27.0)	0.6 (0.2–1.6)		9.3 (5.1–17.0)
	50+	5 (8%)	0.7	6.7 (2.8–16.0)	0.2 (0.1–0.9)		8.1 (2.9–22.8)
	*p*_trend_			<0.001	0.04		0.03
Time since 5-year survival (years)^b^	0–9	25 (39%)	0.8	32.8 (22.2–48.6)	1.0 (ref)		4.3 (2.9–6.4)
	10–19	16 (25%)	0.9	17.2 (10.5–28.0)	0.6 (0.3–1.2)		4.1 (2.4–6.8)
	20–29	16 (25%)	0.9	17.5 (10.8-28.6)	0.7 (0.3–1.4)		7.1 (4.2–11.9)
	30+	7 (11%)	1.1	6.2 (3.0–13.1)	0.2 (0.1–0.6)		5.1 (2.1–12.3)
	*p*_trend_			<0.001	0.01		0.29
Radiotherapy for childhood cancer^c,d^	Yes	48 (86%)	1.4	33.5 (25.3–44.5)	4.9 (1.9–12.7)		9.9 (7.4–13.2)
	No	5 (9%)	0.7	6.7 (2.8–16.1)	1.0 (ref)		1.6 (0.6–4.5)
	Unknown	3 (5%)	–	–	–		–
	*p*_heterogeneity_			<0.001	<0.001		<0.001
Chemotherapy for childhood cancer^c,d^	Yes	37 (66%)	1.0	36.0 (26.1–49.7)	1.0 (0.4–2.3)		8.0 (5.8–11.2)
	No	17 (30%)	1.1	15.6 (9.7–25.1)	1.0 (ref)		5.8 (3.5–9.6)
	Unknown	2 (4%)	–	–	–		–
	*p*_heterogeneity_			0.003		0.93	0.28

*Pyrs* person-years, *Obs* observed, *Exp* expected, *SIR* standardised incidence ratio, *RR* relative risk, *AER* absolute excess risks, *HL* Hodgkin lymphoma, *RT* radiotherapy. *Survivors were exposed to radiotherapy.

^a^RRs were derived from a model including sex, childhood cancer diagnosis, country, decade of childhood diagnosis, age at childhood diagnosis, and attained age.

^b^RRs were derived from a model including sex, childhood cancer diagnosis, country, decade of childhood diagnosis, age at childhood diagnosis, and follow-up time.

^c^RRs were derived from a model including sex, country, age at childhood diagnosis, attained age and treatment.

^d^Excluded Nordic countries (Denmark, Sweden, Norway, Finland, Iceland) and Italy population-based data because of lack of treatment data.

In multivariable analyses, treatment with radiotherapy increased the RR of a salivary gland SPN 4.9-fold (95% CI: 1.9–12.7) compared to treatment without radiotherapy. Treatment with chemotherapy did not seem to increase the RR of developing a salivary gland SPN (RR = 1.0, 95% CI: 0.4–2.3). The SIRs declined with increasing attained age and time since 5-year survival but remained elevated beyond 30 years from 5-year survival (SIR = 6.2, 95% CI: 3.0–13.1).

### Risk of tongue cancer

In all, 26% of all SPNs were located in the tongue (*N* = 38) with all but one type being squamous cell carcinoma (1 spindle cell carcinoma). The risk of developing a tongue SPN was 5.9-fold greater than expected (95% CI: 4.7–7.4) (Table 5). Leukaemia survivors were at greatest risk of developing tongue SPNs (SIR = 25.7, 95% CI: 16.0–41.4), followed by bone sarcoma survivors (SIR = 14.3, 95% CI: 6.4–31.7). Treatment with radiotherapy did not seem to increase the risk (RR = 1.0, 95% CI: 0.4–2.4), but previous treatment with chemotherapy did substantially (SIR = 15.9, 95% CI: 10.6–23.7; RR = 5.6, 95% CI: 1.0–31.2).

**Table 5:** SIR, RR and AER for subsequent primary malignant neoplasms (SPNs) of the tongue by different factors among all cancer survivors in the PanCareSurFup cohort.

SPN on tongue	Level	Obs (%)	Exp	SIR (95% CI)	RR (95%CI)	AER (95% CI)
	Overall	38 (100%)	6.4	5.9 (4.7–7.4)	–	2.5 (1.9–3.3)
Sex^a^	Male	24 (63%)	4.2	5.7 (3.8–8.5)	1.0 (ref)	2.9 (1.8–4.8)
	Female	14 (37%)	2.2	6.5 (3.8–10.9)	0.9 (0.4–1.7)	2.0 (1.1–3.7)
	*p*_heterogeneity_			0.70	0.65	0.34
Type of childhood cancer^a^	Leukaemia	17 (45%)	0.7	25.7 (16.0–41.4)	1.4 (0.5–3.9)	6.3 (3.9–10.4)
	Hodgkin lymphoma	2 (5%)	0.7	3.1 (0.8–12.2)	0.3 (0.1–1.4)	1.4 (0.2–10.8)
	Non-HL	0 (0%)	0.4	–	–	–
	CNS tumour	0 (0%)	1.5	–	–	–
	Neuroblastoma	1 (3%)	0.2	5.2 (0.7–36.8)	0.6 (0.1–5.0)	1.3 (0.1–14.8)
	Retinoblastoma	1 (3%)	0.4	2.6 (0.4–18.3)	0.5 (0.0–4.3)	0.9 (0.0–21.4)
	Wilms tumour	2 (5%)	0.4	5.0 (1.3–20.0)	0.5 (0.1–2.9)	1.5 (0.3–8.3)
	Bone sarcoma	6 (16%)	0.4	14.3 (6.4–31.7)	1.4 (0.4–4.6)	9.8 (4.2–23.2)
	Soft tissue sarcoma	5 (13%)	0.6	8.7 (3.6–21.0)	1.0 (ref))	4.8 (1.8–12.9)
	Other	4 (11%)	1.1	3.7 (1.4–9.8)	0.4 (0.1–1.6)	1.6 (0.4–6.0)
	*p*_heterogeneity_			<0.001	<0.001	<0.001
Decade of childhood cancer diagnosis^a^	<1970	5 (13%)	3.4	1.5 (0.6–3.5)	1.0 (ref)	0.5 (0.0–8.3)
	1970–1979	13 (34%)	1.7	7.4 (4.3–12.8)	2.6 (0.8–8.8)	3.2 (1.7–6.0)
	1980–1989	16 (42%)	1.0	16.5 (10.1–27.0)	3.7 (0.9–14.9)	3.8 (2.2–6.3)
	1990–2008	4 (11%)	0.2	16.0 (6.0–42.6)	2.2 (0.4–12.9)	1.9 (0.7–5.3)
	*p*_trend_			<0.001	0.35	0.07
Age at childhood cancer (years)^a^	0–3	9 (24%)	1.4	6.6 (3.4–12.7)	1.0 (ref)	1.7 (0.8–3.8)
	4–7	6 (16%)	1.1	5.4 (2.4–12.1)	0.9 (0.3-2.6)	1.8 (0.7-4.7)
	8–11	13 (34%)	1.3	9.8 (5.7–17.0)	2.3 (0.8–6.8)	5.6 (3.0–10.2)
	12–21	10 (26%)	2.6	3.8 (2.1–7.1)	1.1 (0.3–4.5)	2.2 (0.9–5.0)
	*p*_trend_			0.36	0.65	0.30
Attained age (years)^a^	<20	2 (5%)	0.1	35.6 (8.9–142.3)	1.0 (ref)	0.5 (0.1–2.0)
	20–29	14 (37%)	0.7	20.8 (12.3–35.1)	0.6 (0.1–2.8)	3.2 (1.8–5.5)
	30–39	11 (29%)	1.3	8.8 (4.9–15.9)	0.3 (0.1–1.4)	3.7 (1.9–7.2)
	40–49	7 (18%)	1.9	3.6 (1.7–7.6)	0.2 (0.0–1.0)	4.2 (1.5–11.7)
	50+	4 (11%)	2.5	1.6 (0.6–4.3)	0.2 (0.0–1.2)	2.9 (0.2–38.1)
	*p*_trend_			<0.001	0.01	0.003
Time since 5-year survival (years)^b^	0–9	7 (18%)	0.4	20.0 (9.5–41.9)	1.0 (ref)	1.2 (0.5–2.6)
	10–19	12 (32%)	0.9	13.0 (7.4–22.9)	0.5 (0.2–1.4)	3.0 (1.6–5.5)
	20–29	10 (26%)	1.6	6.2 (3.3–11.5)	0.3 (0.1–0.9)	3.9 (1.9–8.3)
	30+	9 (24%)	3.5	2.6 (1.3–4.9)	0.3 (0.1–1.2)	4.8 (1.6–13.9)
	*p*_trend_			<0.001	0.07	0.02
Radiotherapy for childhood cancer^c,d^	Yes	18 (64%)	2.9	6.3 (4.0–10.0)	1.0 (0.4–2.4)	3.2 (1.9–5.6)
	No	8 (29%)	1.4	5.7 (2.8–11.4)	1.0 (ref)	2.5 (1.1–5.8)
	Unknown	2 (7%)	–	–	–	–
	*p*_heterogeneity_			0.80	0.92	0.62
Chemotherapy for childhood cancer^c,d^	Yes	24 (86%)	1.5	15.9 (10.6–23.7)	5.6 (1.0–31.2)	5.0 (3.3–7.7)
	No	2 (7%)	2.6	0.8 (0.2–3.1)	1.0 (ref)	–
	Unknown	2 (7%)	–	–	–	–
	*p*_heterogeneity_			<0.001	0.03	<0.001

*Pyrs* person-years, *Obs* observed, *Exp* expected, *SIR* standardised incidence ratio, *RR* relative risk, *AER* absolute excess risks, *HL* Hodgkin lymphoma.

^a^RRs were derived from a model including sex, childhood cancer diagnosis, country, decade of childhood diagnosis, age at childhood diagnosis, and attained age.

^b^RRs were derived from a model including sex, childhood cancer diagnosis, country, decade of childhood diagnosis, age at childhood diagnosis, and follow-up time.

^c^RRs were derived from a model including sex, country, age at childhood diagnosis, attained age and treatment.

^d^Excluded Nordic countries (Denmark, Sweden, Norway, Finland, Iceland) and Italy population-based data because of lack of treatment data.

## Discussion

### Main findings

This cohort study with almost 70,000 childhood cancer survivors is the largest study to date to investigate the risk of oral SPNs among childhood cancer survivors. Our findings show that childhood cancer survivors are at 5-fold risk of developing oral SPNs compared to general population and that even after age 40 years the risk remains 4-fold higher than expected. Exposure to previous radiotherapy increases the risk of salivary glands SPN substantially, particularly among leukaemia and Hodgkin lymphoma survivors. A novel finding includes the identification of a substantially increased risk of tongue SPNs following exposure to chemotherapy.

### Risk of salivary gland malignancies

Previous studies have shown that exposure to radiation, particularly during childhood, plays a role in the development of salivary gland tumours with evidence mostly stemming from the atomic bomb survivor study [15, 24, 25]. The most recent publication from the atomic-bomb survivor study suggested a strong radiation dose-response for salivary gland tumours, but no evidence of a radiation effect for other oral cancer types [24, 26]. Most previous studies included few salivary gland malignancies, i.e., 50 cases among atomic bomb studies [26], and fewer among most studies investigating the risk of salivary gland malignant neoplasms following therapeutic radiation for paediatric malignancies [14, 15] or other conditions [27]. Within the North-American Childhood Cancer Survivor Study (CCSS) 23 salivary SPNs were reported [15] with an SIR of 39 (95% CI: 25.4–57.8). When comparing the SIR in our study with that of the CCSS using the same childhood cancer diagnosis period, the SIR of a salivary gland SPN was 22-fold (95% CI: 14.1–34.7), which is somewhat lower but not inconsistent. The CCSS study suggested that the risk is elevated for the first two decades after childhood cancer treatment, but here we found that the risk is elevated at least for three decades and well beyond age 50 years. The risk was especially high among leukaemia and Hodgkin lymphoma survivors, likely because the salivary glands were within the radiation fields of prophylactic or therapeutic cranial irradiation, total body irradiation (TBI), or the neck and Waldeyer’s ring, or due to potential radiation scatter from other irradiated sites.

### Risk of tongue malignancies

To our knowledge, risks of tongue carcinomas in childhood cancer survivors have not been reported before, except for the mentioning of three cases in the CCSS [12] and in a case report in a patient of AML [28]. We report here, for the first time, that survivors of childhood cancer, mostly leukaemia and bone sarcoma survivors, are at substantial risk of developing squamous cell carcinoma of the tongue following exposure to chemotherapy. Three-quarters of the tongue SPNs occurred under age 40 years which is unusual as in the general population squamous cell carcinoma of the tongue generally occurs at much older ages and is typically caused by chronic smoking and/or alcohol exposure. There are number of possible explanations for the increased risk following chemotherapy. Those who received HSCT are more likely to have been treated with aggressive chemotherapy followed by prolonged immunosuppression and may also be more prone to develop chronic graft-versus-host disease [29, 30]. It may also be that chemotherapy may impair the ability of the immune system to target mutagenic cells and fight infections such as HPV—a well-known cause of squamous cell carcinoma of the tongue [31, 32]. In terms of chemotherapy in general, another factor that might promote the development of squamous cell carcinoma of the tongue is chronic inflammation due to cancer treatment [33, 34]. To our knowledge, only a small number of previous studies reported squamous cell carcinomas of the tongue in recipients of HSCT, mostly for leukaemia, but the total number of subsequent tongue malignancies reported in the literature was small [29, 30, 35]. Lastly, a genetic predisposition—such as TP53 mutations—may be implicated in developing of tongue cancer. There is a possibility that the association we observed between being treated with chemotherapy and risk of tongue cancer is not causative but due to underlying cancer genetic predisposition in those survivors treated with chemotherapy, such as bone and soft-tissue sarcoma patients. Fanconi anaemia may also increase the risk of a tongue SPN, mainly among acute myeloid leukaemia (AML) survivors, but only three tongue SPNs were among AML survivors suggesting this is an unlikely explanation [36]. A case–control study may be able to address the risks related with specific treatments, life-style factors, and genetic factors.

### Clinical implications

Although the absolute risk of developing oral cancer is low for childhood cancer survivors, survivors treated with direct radiotherapy to the head and neck or radiotherapy involving potential radiation scatter to the head and neck areas are at risk of developing salivary gland SPNs. Those treated with chemotherapy are at risk of developing tongue SPNs. It has been recommended that survivors discuss their cancer history with their dentists and visit a dentist at least yearly and preferable every 6 months [37]. It would be important for health professionals, including dentists, dental hygienists, and otorhinolaryngologists, responsible for the care of such survivors to be aware of the treatment patients received historically and the associated risks so that any oral cancers can be detected early.

### Study limitations

A potential limitation of our study may include the lack of detailed treatment information such as type and cumulative dose of chemotherapy agents, and cumulative radiation doses and radiation fields. Collecting such detailed treatment information on nearly 70,000 individuals, with a substantial proportion of survivors treated several decades ago, within this Pan-European cohort would be practically not feasible.

Another potential limitation of the study is that we were unable to control for potential confounding factors such as smoking and alcohol intake and thus some of the observed effects may be attributable to smoking and drinking. However, survivors of childhood cancer are generally less likely to smoke and drink than the general population [38]; hence, if there were to be potential confounding by such lifestyle factors this would inflate the already substantial risks. Moreover, previous studies have suggested that the effect of smoking and alcohol on the association between radiation exposure and subsequent oral cancer risk is minimal [26].

## Conclusions

Although the absolute risk of developing an oral SPN is low, survivors are at 5-fold increased risk of developing oral SPNs compared to the general population. Exposure to previous radiotherapy increases the risk of salivary gland SPNs substantially, whilst exposure to chemotherapy increases the risk of tongue SPNs substantially. Communication between general practitioners, oncologists, and dentists and increased awareness of the risk among both health care professionals and survivors may play a crucial role in identifying and treating oral SPNs early.

## Supplementary information

Supplementary Tables

Checklist_AJ

## Data Availability

Access to anonymised data may be granted under conditions agreed with the relevant (local) legal and research ethics committees and with appropriate data-sharing agreements and permissions from each data provider in place. Any data sharing would have to comply with the EU General Data Protection Regulation. The data that support the findings of this study are not publicly available due to privacy and ethical restrictions. Aggregated data in the form of tables may be available on reasonable request.

## Code availability

Code is available upon reasonable request by contacting the corresponding author.
