# Supplementary material for: Risk of subsequent primary oral cancer in a cohort of 69,460 5-year survivors of childhood and adolescent cancer in Europe: the PanCareSurFup study
Source: Br J Cancer. 2022 Nov 1;128(1):80–90. doi: 10.1038/s41416-022-02016-w (PMC9814398; doi:10.1038/s41416-022-02016-w)
Supplement: Supplementary file 1 — Supplementary Tables [file 41416_2022_2016_MOESM1_ESM.docx]

# Supplementary

Table.S.1. Cohort characteristics of data providers to the PanCareSurFup study

| **Cohort** | **No. of 5-year Survivors** | **Period of Diagnosis** | **Study Design** | **Age at childhood cancer** | **Study exit date** | **% Loss to Follow-Up** |
| --- | --- | --- | --- | --- | --- | --- |
| France | 3,138 | 1946-1986 | Hospital-based | 0-18 | Sep-14 | - |
| Hungary | 4,885 | 1971-2008 | Population-based | 0-19 | Dec-14 | - |
| Italy (PB) | 7,476 | 1964-2005 | Population-based | 0-19 | May-10 | 2 |
| Italy (HB) | 1,490 | 1960-2008 | Hospital-based | 0-19 | Dec-12 | - |
| Netherlands | 6,044 | 1963-2001 | Population-based | 0-17 | Dec-12 | 6 |
| Denmark | 4,840 | 1943-1998 | Population-based | 0-19 | Dec-03 | 5 |
| Sweden | 7,709 | 1958-1998 | Population-based | 0-19 | Dec-03 | 2 |
| Norway | 3,783 | 1953-1997 | Population-based | 0-19 | Dec-02 | 3 |
| Finland | 6,229 | 1953-2006 | Population-based | 0-19 | Dec-11 | 5 |
| Iceland | 275 | 1955-1998 | Population-based | 0-19 | Dec-03 | 5 |
| Slovenia | 1,252 | 1960-2002 | Population-based | 0-16 | Jul-14 | 5 |
| Switzerland | 4,379 | 1964-2005 | Population-based | 0-19 | Dec-13 | - |
| UK | 17,960 | 1940-1991 | Population-based | 0-15 | Sep-16 | 5 |
| **Total** | **69,460** | **1940-2008** |  | **0-19** |  |  |

*PB; population-based, HB; hospital-based, UK; United Kingdom*

Table.S.2. Table of codes for different revisions of the International Classification of Diseases (ICD)

| **Malignant Neoplasm** | **ICD-10** | **ICD-9** | **ICD-8** | **ICD-7** |
| --- | --- | --- | --- | --- |
| Lip | C00.0-C00.9 | 140.0-140.9 | 140.0-140.9 | 140.0-140.9 |
| Tongue | C01.0-C02.9 | 141.0-141.9 | 141.0-141.9 | 141.0-141.9 |
| Oral cavity | C03.0-C06.9 | 143.0-145.9 | 143.0-145.9 | 143.0-145.9 |
| Salivary glands | C07.0-C08.9 | 142.0-142.9 | 142.0-142.9 | 142.0-142.9 |
| Pharynx | C09.0-C13.9,  C14.0 and C14.2 | 146.0-149.1 | 146.0-149.1 | 146.0-148.9 |
| Overall | C00.0-C14.8 | 140.0-149.9 | 140.0-149.9 | 140.0-148.9 |
